# The Eleventh International Medical Congress

**Published:** 1893-02

**Authors:** A. Jacobi

**Affiliations:** Chairman of the American National Committee


### THE ELEVENTH INTERNATIONAL MEDICAL CONGRESS. SECTION OF ODONTOLOGY.

To the Editor of the International Dental Journal:

Sir,—The Eleventh International Medical Congress will be held in Rome, 1893, beginning the 24th of September and continuing until the 1st of October.

The Committee on Organization, following the precedent established in London, 1881, has provided for a Section of Odontology. As America has contributed pre-eminently to the scientific progress of dental surgery, it is hoped that the dental profession in America will be creditably represented: all reputable practitioners are entitled to membership in that section.

The time chosen is the most delightful of all the year, and to those who have never visited the "Eternal City" the meeting of the Congress will afford a rare opportunity.

The North German Lloyd Steamship Company has an established line of first-class steamers to Genoa, making the passage in less than eleven days. It proposes to reduce the fare to Genoa by twenty per cent., and the return-trip by ten per cent., to those attending the Congress.

The French Railway Company has also offered a reduction of fifty per cent. on its fare.

Dr. Norman W. Kingsley, 115 Madison Avenue, New York, has been appointed Member of the American National Committee for the Promotion of the Interests of the Odontological Section. All communications in reference to that section should be addressed to him.

A. Jacobi, M.D.,
*Chairman of the American National Committee.*

